# Medical Research Council-sumscore: a tool for evaluating muscle weakness in patients with post-intensive care syndrome

**DOI:** 10.1186/s13054-020-03282-x

**Published:** 2020-09-18

**Authors:** Zeynep Turan, Mahir Topaloglu, Ozden Ozyemisci Taskiran

**Affiliations:** Department of Physical Medicine and Rehabilitation, Koc University School of Medicine, Maltepe Mah, Davutpasa Cad, No:4, Topkapı, Zeytinburnu, 34010 Istanbul, Turkey

Dear Editor,

COVID-19 may lead to severe acute respiratory distress syndrome requiring intensive care unit (ICU) support. Patients surviving respiratory distress could develop post-intensive care syndrome (PICS) that includes ICU-acquired weakness (ICUAW). Nearly 66% of COVID-19 patients have clinically important muscle weakness following discharge [1]. Therefore, communication between the critical care and rehabilitation physician is important to evaluate the physical function of COVID-19 survivors to start rehabilitation timely.

The comprehensive examination of muscle strength in COVID-19 is not easy. Muscle strength can be evaluated by manual muscle testing and dynamometer. Electrophysiological study is important in diagnosing critical illness neuromyopathy; however, its correlation with muscle weakness is not clear. Ultrasonography can detect atrophy and structural changes but does not correlate with muscle function [2].

Medical Research Council (MRC)-sumscore evaluates global muscle strength. Manual strength of six muscle groups (shoulder abduction, elbow flexion, wrist extension, hip flexion, knee extension, and ankle dorsiflexion) is evaluated on both sides using MRC scale. Summation of scores gives MRC-sumscore, ranging from 0 to 60. This score was developed for detecting early strength alterations in patients with Guillain-Barré syndrome, especially who were bedridden and receiving artificial ventilation. The sensitivity and interobserver agreement of MRC-sumscore was demonstrated [3]. Despite its ceiling effect, this score reliably identifies significant weakness (< 48) and even better in severe weakness (< 36) [4] which is the main medical interest for treatment in ICUAW.

Handgrip strength is a rapid, simple, and objective tool that is measured by handheld dynamometer represents global muscle strength. The cutoff value for handgrip strength in critically ill patients is defined as < 11 kg force for males and < 7 kg force for females which is below that of the age- and sex-matched patients [5]. It was proposed as an alternative to MRC in ICUAW [5]. However, examination of other muscles by MRC-sumscore might give additional information since the neurological consequences of COVID-19 are not clear yet. ICUAW is more pronounced in proximal muscles; therefore, direct evaluation of proximal muscles is also valuable. MRC is associated with mortality, hospital, and ICU-free days in ICUAW more strongly than handgrip strength [5].

In conclusion, MRC-sumscore is a valid, reliable, objective, and easy method to evaluate the global muscle strength including PICS related to COVID-19. It provides beneficial information about the clinical course. Its bedside applicability without necessitating any device makes MRC-sumscore a valuable tool in the follow-up of patients with PICS.

## Data Availability

Not applicable

## Abbreviations

ICU: Intensive care unit
ICUAW: Intensive care unit acquired weakness
MRC: Medical Research Council
PICS: Post-intensive care syndrome
